# Relation of High CHA₂DS₂-VASc Score With Contrast-Induced Nephropathy Following Percutaneous Coronary Intervention

**DOI:** 10.7759/cureus.107528

**Published:** 2026-04-22

**Authors:** Hashan Mamunur Rahman, Mohammad Ullah, Md. Saifur Rahman, Dewan Mushfiq Raihan

**Affiliations:** 1 Emergency Department, National Institute of Cardiovascular Diseases, Dhaka, BGD; 2 Cardiology Department, National Institute of Cardiovascular Diseases, Dhaka, BGD; 3 Cardiology Department, Shajahanpur Upazilla Health Complex, Bogura, BGD

**Keywords:** acute coronary syndrome (acs), cha₂ds₂-vasc score, chronic coronary syndrome (ccs), contrast-induced nephropathy (cin), percutaneous coronary intervention (pci)

## Abstract

Background

Contrast-induced nephropathy (CIN) is a prevalent and significant complication after percutaneous coronary intervention (PCI), leading to heightened morbidity, mortality, and healthcare expenditures. Recognizing high-risk patients prior to the treatment is essential, particularly in resource-constrained environments such as Bangladesh. Numerous global research studies have indicated that the CHA₂DS₂-VASc, which comprises easily accessible clinical factors, may function as an intuitive prediction tool for CIN. Nevertheless, information concerning this association is scarce within the Bangladeshi context. Therefore, this study aimed to determine the relationship between high CHA₂DS₂-VASc scores and the development of CIN following PCI, and to evaluate the predictive value of this score.

Methods

This prospective observational study took place at Dhaka Medical College Hospital (DMCH), Dhaka, Bangladesh, from July 2023 to July 2024. Patients who underwent PCI in the Department of Cardiology comprised the study population. A total of 104 patients with acute coronary syndrome (ACS) and chronic coronary syndrome (CCS) were recruited according to the selection criteria by convenience sampling. Participants were categorized into two groups based on their baseline CHA₂DS₂-VASc scores: Group A (score ≥2) and Group B (score <2), each including 52 patients. The occurrence of CIN, defined as an absolute rise in serum creatinine greater than 0.5 mg/dL or a relative increase of at least 25% from baseline within 48 to 72 hours after the procedure, was the primary outcome evaluated. The DMC's institutional review board granted approval for the study to proceed, subject to ethical considerations.

Results

CIN developed in 13.5% of the patients in this study. The incidence of CIN was significantly higher among patients with a CHA₂DS₂-VASc score ≥2 compared to those with a CHA₂DS₂-VASc score <2 (21.2% vs. 5.8%, p-value: 0.022). CIN showed significant associations with prior use of angiotensin receptor blockers (ARBs) and a CHA₂DS₂-VASc score ≥2. However, no statistically significant association was observed between CIN and the volume of contrast administered during the procedure. In multivariate logistic regression analysis, only a CHA₂DS₂-VASc score ≥2 remained an independent predictor of CIN (adjusted odds ratio (AOR) = 5.75; 95% confidence interval (CI): 1.663-19.92; p-value: 0.006), indicating that patients with higher CHA₂DS₂-VASc scores had more than fivefold increased odds of developing CIN. CHA₂DS₂-VASc score ≥2 had moderate sensitivity (78.57%) and low specificity (54.44%) for predicting CIN. It had a low positive predictive value but a high negative predictive value, indicating it was good for ruling out CIN and should be used as a screening tool rather than a confirmatory tool.

Conclusion

The development of CIN after PCI was significantly associated with a higher CHA₂DS₂-VASc score. Patients with a score ≥2 had significantly increased odds of developing CIN, and this remained an independent predictor. Moreover, the CHA₂DS₂-VASc score ≥2 is a simple and practical tool for early identification of patients at higher risk of CIN prior to PCI; it is particularly useful for ruling out CIN and is better suited as a screening rather than a confirmatory tool.

## Introduction

Globally, coronary artery disease (CAD) continues to be the most prevalent cause of mortality and disability, resulting in the highest number of disability-adjusted life years (DALYs) lost. The burden is especially severe in low- and middle-income countries (LMICs), where access to comprehensive cardiac care is usually limited [1]. Percutaneous coronary intervention (PCI) has emerged as a critical therapeutic approach in the treatment of CAD, as it has been shown to substantially improve myocardial perfusion, alleviate symptoms, and improve survival outcomes [2]. Nevertheless, PCI is not without its hazards, despite its established advantages. One of the most serious consequences is the deterioration in renal function following the injection of iodinated contrast agents, a condition commonly recognized as contrast-induced nephropathy (CIN) [3]. While numerous instances of CIN tend to resolve on their own, more severe cases have the potential to advance to chronic kidney disease, necessitate dialysis, and significantly elevate mortality rates. Individuals with prior renal dysfunction or various coexisting health conditions face heightened risks, often enduring extended hospitalizations, experiencing more intricate clinical courses, and encountering increased occurrences of adverse cardiovascular events. The emergence of CIN following cardiac procedures significantly elevates mortality rates, presenting a two- to fivefold increase when juxtaposed with patients who do not experience CIN [4,5]. CIN develops in nearly 20% of patients undergoing primary PCI and is associated with increased morbidity, prolonged hospital stays, and higher healthcare costs [6,7]. Its incidence varies from 7% to 25% depending on the risk profile of the population [8-10]. Therefore, early risk stratification is essential to identify high-risk patients and initiate appropriate preventive measures before exposure to contrast media. It is essential to foresee the risk of CIN, especially when it allows for early detection before a procedure. This facilitates the implementation of enhanced preventive measures for those at elevated risk, before, during, and subsequent to exposure to contrast media [11].

Although various hazards associated with CIN have been identified, a thorough understanding of their cumulative effects remains limited. Mehran and associates developed the Mehran score, a crucial prediction tool for assessing the likelihood of CIN in patients following PCI. This score has subsequently been acknowledged as the standard in the field [12]. The Mehran score is typically employed post-PPCI, thereby postponing the evaluation of CIN risk. A recent study indicated that the neutrophil-to-lymphocyte ratio (NLR), a straightforward yet potent parameter, may predict CIN after PCI [13]. Recent studies have examined the use of CHA₂DS₂-VASc as a preprocedural predictor of CIN, offering a significant alternative to traditional grading systems such as the Mehran score [11,14]. An elevated CHA₂DS₂-VASc score is associated with a heightened risk of CIN [15]. The CHA₂DS₂-VASc score has proven effective in forecasting CIN in individuals with acute myocardial infarction undergoing angiography [11,16-20].

In Bangladesh, the rising burden of CAD has increased reliance on PCI, where CIN remains a serious complication. Limited access to routine renal assessment, inconsistent preventive practices, and costly renal support make early prediction of CIN crucial, especially in the context of the increasing number of patients undergoing PCI in Bangladesh who are at risk for this complication. The Mehran score, though widely used, depends on post-procedural factors and is less practical in emergency settings common in Bangladesh, where timely assessment is critical for patient outcomes. The CHA₂DS₂-VASc score, based on readily available pre-procedural clinical variables, may provide a simple, cost-effective tool for CIN risk prediction. Validating this score locally could improve early risk stratification, guide prevention, and optimize healthcare resource utilization.

The primary objective was to determine the relationship between the higher CHA₂DS₂-VASc scores and the development of CIN following PCI. The secondary objective was to evaluate the predictive value of the score using a predefined threshold (CHA₂DS₂-VASc score ≥2).

## Materials and methods

A prospective observational study was performed at the Department of Cardiology, Dhaka Medical College (DMC), from July 2023 to July 2024. The study received ethical approval from the institutional review board of DMC (Ref: ERC/DMC/ECC//2023/189), and informed written consent was obtained from the participants.

The study population comprised all patients scheduled for PCI in the cardiology department. A total of 104 patients over 18 years of age, of both genders, with acute coronary syndrome (ACS) and chronic coronary syndrome (CCS) who underwent PCI were included in the study using a convenience sample method. Patients with renal impairment (serum creatinine >1.4 mg/dL), anemia (hemoglobin <10 g/dL), chronic total occlusion (CTO), multivessel disease, infection or inflammatory conditions, recent exposure to radiographic contrast media (within 10 days of enrollment), cardiogenic shock, and the use of nephrotoxic agents such as non-steroidal anti-inflammatory drugs (NSAIDs), aminoglycosides, amphotericin B, and vancomycin were excluded from the study. Data were collected using a pre-tested data collection form that included sociodemographic variables, anthropometric measurements, history of comorbidity (hypertension (HTN), diabetes mellitus (DM), and dyslipidemia), personal habits (smoking), CHA₂DS₂-VASc score, and development of CIN.

Assessment of CHA₂DS₂-VASc score

At baseline, the CHA₂DS₂-VASc score was calculated for each patient by assigning a score of 1 to the following variables: (a) chronic heart failure (CHF) or left ventricular systolic dysfunction with ejection fraction (EF) ≤40%, (b) HTN, (c) age 65-74 years, (d) DM, (e) vascular disease, and (f) female gender. Additionally, 2 points were assigned for (g) age 75 years or older and (h) a history of stroke or transient ischemic attack [16]. A score of 1 was allocated to each patient due to the presence of CAD caused by vascular atherosclerosis, requiring a PCI. All PCI operations were performed by skilled interventional cardiologists utilizing either the transfemoral or transradial technique, determined by proficiency and technical viability. Participants were classified into two groups based on the CHA₂DS₂-VASc score: Group A (CHA₂DS₂-VASc score ≥2) and Group B (CHA₂DS₂-VASc score <2), with at least 52 patients in each group.

Outcome measurement (CIN)

This study primarily focused on the development of CIN following PCI procedures. CIN was defined as a serum creatinine elevation beyond 0.5 mg/dL or a relative rise of 25% or more from baseline, occurring within 48 to 72 hours post-procedure. Serum creatinine levels were assessed 24 hours before and 48 hours following PCI, utilizing an automated analyzer Erba Mannheim XL-1000 (Erba Lachema s.r.o., Brno, Czech Republic).

Statistical analysis

Data were analyzed using IBM SPSS Statistics for Windows, Version 26 (Released 2019; IBM Corp., Armonk, New York). Continuous data were presented as mean ± standard deviation (SD), whereas categorical variables were expressed as frequency and percentage. The Shapiro-Wilk test was employed to assess the normality of continuous data distributions. Comparisons of continuous variables between groups were performed using the independent samples t-test, and categorical variables were evaluated using the chi-square test. Comparisons of serum creatinine levels before and after the procedure within groups were conducted using the Wilcoxon signed-rank test. A binary logistic regression analysis was performed to identify predictors of CIN. Initially, univariate analysis was conducted to determine the crude odds ratio (COR) and 95% confidence interval (CI) for each potential risk factor. Variables with a p-value < 0.05 in univariate analysis were included in a multivariate logistic regression model to identify independent predictors of CIN, with results reported as adjusted odds ratios (AOR) and 95% CIs. Additionally, the diagnostic performance of the CHA₂DS₂-VASc score ≥2 for predicting CIN was evaluated by calculating sensitivity, specificity, positive predictive value (PPV), negative predictive value (NPV), and overall accuracy. A p-value <0.05 was considered statistically significant for all analyses.

## Results

Patients in Group A were significantly older compared to those in Group B (mean age: 60.64 ± 7.15 vs. 52.48 ± 9.88 years, p-value: 0.001), with a greater proportion aged ≥60 years (59.6% vs. 34.6%, p-value: 0.003). Gender distribution was similar between the groups (male: 67.3% vs. 73.1%, p-value: 0.52). HTN (86.5% vs. 25.0%, p-value: < 0.001) and DM (46.2% vs. 17.3%, p-value: 0.002) were significantly more prevalent in Group A, whereas smoking, dyslipidemia, and family history of CAD did not differ significantly. In terms of medication use, angiotensin-converting enzyme (ACE) inhibitor (50.0% vs. 5.8%, p-value: < 0.001), angiotensin II receptor blocker (ARB) (36.5% vs. 15.4%, p-value: 0.014), and metformin use (38.5% vs. 11.5%, p-value: 0.002) were more frequent in Group A, whereas statin use was similar between groups. The distribution of clinical presentation (ACS vs. CCS) did not differ significantly (p-value: 0.539) (Table 1).

**Table 1:** Baseline demographic, clinical, and risk factor characteristics of patients in Group A and Group B ^a ^Chi-square test ^b ^Independent samples t-test Data presented as frequency (%) and mean ± SD Group A: patients with CHA_2_DS_2_-VASc score ≥2; Group B: patients with CHA_2_DS_2_-VASc <2 CAD: Coronary artery disease; ACS: Acute coronary syndrome; CCS: Chronic coronary syndrome; ACE inhibitor: Angiotensin-converting enzyme inhibitors; ARB blocker: Angiotensin II receptor blocker

Characteristics		Group A (n=52)	Group B (n=52)	Total	p-value
Age (years)	<40	0 (0.0)	8 (15.4)	8 (7.7)	^a ^0.003
	40–49	5 (9.6)	7 (13.5)	12 (11.5)	
	50–59	16 (30.8)	19 (36.5)	35 (33.7)	
	60–69	25 (48.1)	18 (34.6)	43 (41.3)	
	≥70	6 (11.5)	0 (0.0)	6 (5.8)	
	Mean ± SD	60.64 ± 7.15	52.48 ± 9.88	56.56 ± 9.51	^b^ 0.001
Gender	Male	35 (67.3)	38 (73.1)	73 (70.2)	^a ^0.520
	Female	17 (32.7)	14 (26.9)	31 (29.8)	
Risk factors	Hypertension	45 (86.5)	13 (25.0)	58 (55.8)	^a ^<0.001
	Diabetes mellitus	24 (46.2)	9 (17.3)	33 (31.7)	^a ^0.002
	Smoking	19 (36.5)	12 (23.1)	31 (29.8)	^a ^0.133
	Dyslipidemia	9 (17.3)	13 (25.0)	22 (21.2)	^a ^0.337
	Family history of CAD	7 (13.5)	8 (15.4)	15 (14.4)	^a ^0.780
Drug history	ACE inhibitor	26 (50.0)	3 (5.8)	29 (27.9)	^a ^<0.001
	ARB blocker	19 (36.5)	8 (15.4)	27 (26.0)	^a ^0.014
	Metformin	20 (38.5)	6 (11.5)	26 (25.0)	^a ^0.002
	Statin	9 (17.3)	13 (25.0)	22 (21.2)	^a ^0.337
Type of CAD	ACS	35 (67.3)	32 (61.5)	67 (64.4)	^a ^0.539
	CCS	17 (32.7)	20 (38.5)	37 (35.6)	

The mean contrast volume used was comparable between Group A and Group B (96.25 ± 14.38 mL vs. 96.15 ± 14.48 mL, p-value: 0.97). Baseline serum creatinine levels before PCI did not differ significantly between the two groups (1.07 ± 0.21 mg/dL vs. 1.08 ± 0.24 mg/dL, p-value: 0.78). However, 48 hours after PCI, serum creatinine was significantly higher in Group A compared to Group B (1.37 ± 0.24 mg/dL vs. 1.27 ± 0.24 mg/dL, p-value: 0.03) (Table 2).

**Table 2:** Comparison of contrast volume and serum creatinine levels between Group A and Group B An independent samples t-test was done. Data presented as mean ± SD Group A: Patients with CHA_2_DS_2_-VASc score ≥2; Group B: Patients with CHA_2_DS_2_-VASc score <2 PCI: Percutaneous coronary intervention

Variable		Group A (n=52)	Group B (n=52)	p-value
Contrast volume (mL)		96.25 ± 14.38	96.15 ± 14.48	0.97
Serum creatinine (mg/dL)	Before PCI	1.07 ± 0.21	1.08 ± 0.24	0.78
	48 hours after PCI	1.37 ± 0.24	1.27 ± 0.24	0.03

**Figure 1:**
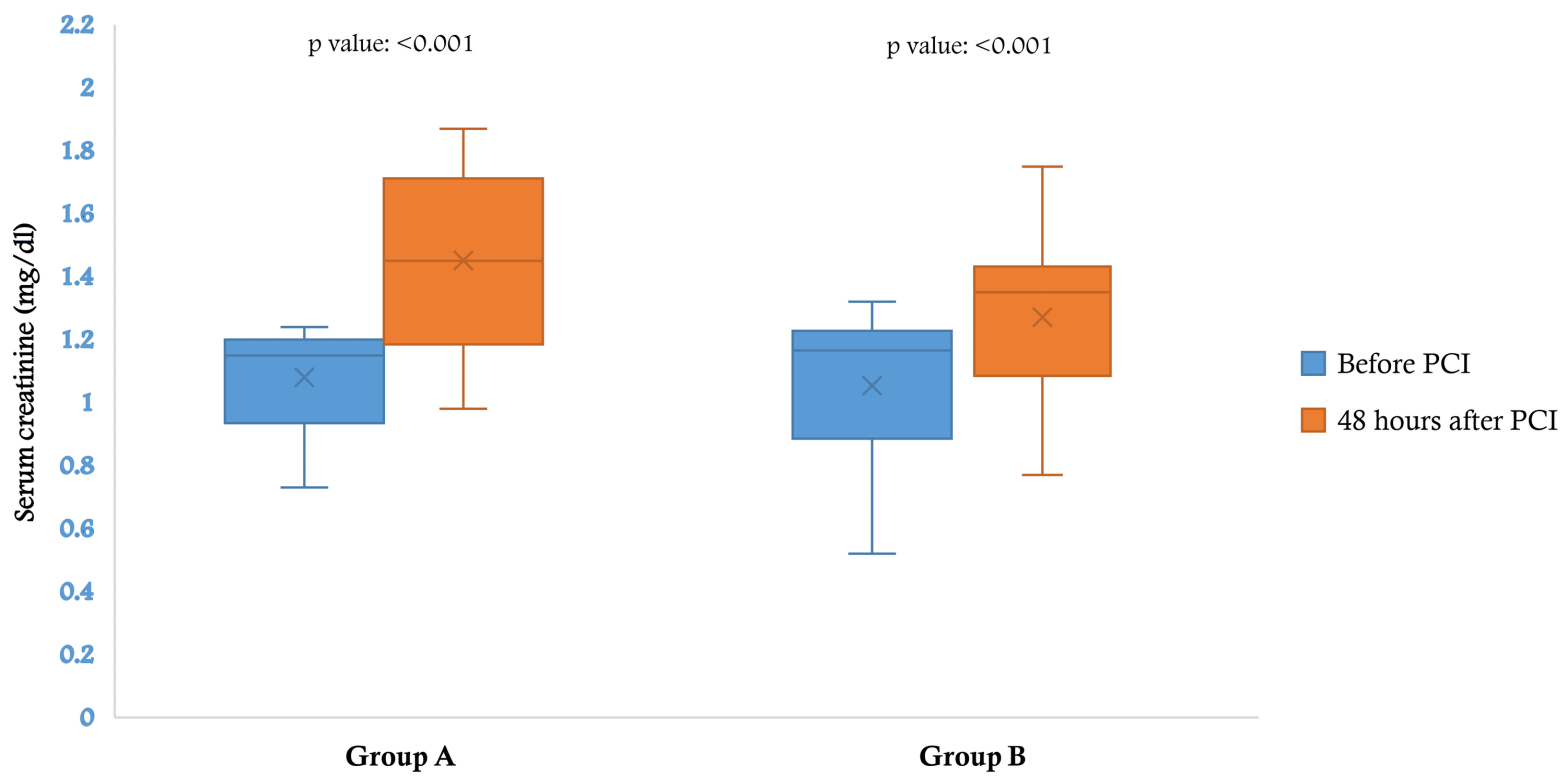
Comparison of serum creatinine levels before and 48 hours after PCI according to CHA₂DS₂-VASc score category Wilcoxon signed-rank test was done Group A: Patients with CHA_2_DS_2_-VASc score ≥2; Group B: Patients with CHA_2_DS_2_-VASc score <2 PCI: Percutaneous coronary intervention

Within-group analysis demonstrated a statistically significant elevation in serum creatinine levels from baseline to 48 hours post-procedure in both groups of patients (p-value: < 0.001). However, the extent of this increment was more pronounced among individuals with a CHA₂DS₂-VASc score ≥ 2, indicating a greater degree of post-procedural renal function deterioration in this subgroup. The wider interquartile range in Group A after PCI indicates greater variability and a higher incidence of renal impairment among patients with CHA₂DS₂-VASc scores ≥ 2 (Figure 1).

**Figure 2:**
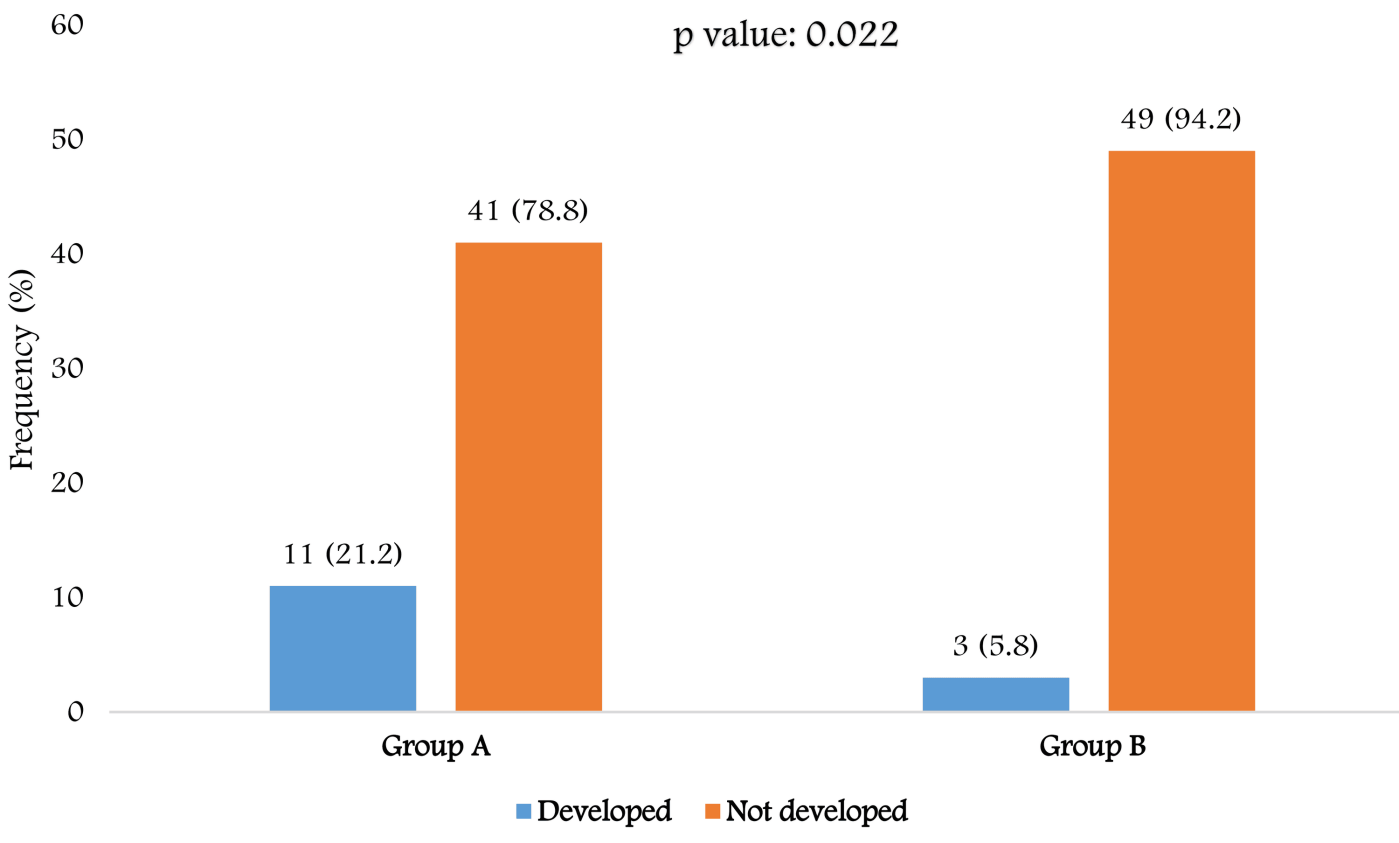
Comparison of contrast-induced nephropathy (CIN) development between Group A and Group B Group A: Patients with CHA_2_DS_2_-VASc score ≥2; Group B: Patients with CHA_2_DS_2_-VASc score <2

Approximately 13.5% of participants developed CIN following PCI. The proportion of CIN was significantly higher in Group A compared to Group B (21.2% vs. 5.8%, p-value: 0.022). In Group A, 11 patients (21.2%) developed CIN while 41 (78.8%) did not. In contrast, only three patients (5.8%) in Group B developed CIN, whereas 49 (94.2%) remained unaffected. This finding suggests that Group A patients were at substantially higher risk of CIN following PCI compared with Group B (Figure 2).

The CHA₂DS₂-VASc score ≥2 demonstrated moderate sensitivity (78.57%) but limited specificity (54.44%) for predicting CIN. Although the PPV was low (21.15%), the high NPV (94.23%) indicates good utility in excluding CIN. The overall accuracy was modest (57.69%), suggesting that the score is better suited as a screening rather than a confirmatory tool in this context (Table 3).

**Table 3:** Diagnostic performance of CHA₂DS₂-VASc score ≥2 for predicting contrast-induced nephropathy (CIN) Data are presented as percentages with 95% confidence intervals

Metric	Value (%)	95% Confidence interval	
		Lower	Upper
Sensitivity	78.57%	49.20%	95.34%
Specificity	54.44%	43.60%	64.98%
Positive predictive value (PPV)	21.15%	15.84%	27.67%
Negative predictive value (NPV)	94.23%	85.48%	97.84%
Accuracy	57.69%	47.61%	67.32%

In this study, univariate logistic regression analysis showed that most variables, including total cholesterol, low-density lipoprotein cholesterol (LDL-C), and ACE inhibitor use, were not significantly associated with the development of CIN (p-value: >0.05). Although ARB use demonstrated a significant association in the unadjusted model (COR=3.20, 95% CI: 2.15-4.12, p-value: 0.043), this association lost statistical significance after adjustment (AOR=3.09, 95% CI: 0.759-12.59, p-value: 0.115), indicating that the initial relationship was likely influenced by confounding factors. In contrast, a CHA₂DS₂-VASc score ≥2 was significantly associated with the development of CIN in both univariate and multivariate analyses. Patients with a CHA₂DS₂-VASc score ≥2 had higher odds of developing CIN in the unadjusted model (COR=4.38, 95% CI: 1.15-16.77, p-value: 0.031), and this association remained statistically significant after adjustment (AOR=5.75, 95% CI: 1.663-19.92, p-value: 0.006) (Table 4).

**Table 4:** Univariate and multivariate logistic regression analysis of risk factors for contrast-induced nephropathy (CIN) following percutaneous coronary intervention (PCI) Univariate and multivariate logistic regression were done. Data presented as odds ratios (OR) with 95% confidence intervals (CI) COR: Crude odds ratio; AOR: Adjusted odds ratio; CI: Confidence interval; LDL-C: Low-density lipoprotein cholesterol; ARB: Angiotensin receptor blocker; ACEi: Angiotensin-converting enzyme inhibitor

Variables	COR	95% CI of COR (Lower–Upper)	p-value	AOR	95% CI of AOR (Lower–Upper)	p-value
Total cholesterol	0.99	0.98–1.01	0.48	-	-	-
LDL-C	0.98	0.96–1.02	0.25	-	-	-
ACEi	0.67	0.17–2.60	0.56	-	-	-
ARB	3.20	2.15–4.12	0.043	3.09	0.759–12.59	0.115
CHA₂DS₂-VASc score ≥ 2	4.38	1.15–16.77	0.031	5.75	1.663–19.92	0.006

## Discussion

CIN is a significant complication that can result in heightened morbidity and death in individuals undergoing PCI for ACS [21]. CIN is the third most significant cause of hospital-acquired acute kidney injury (AKI), following hypoperfusion (or prerenal damage) and postoperative AKI [8]. CIN was associated with in-hospital HTN and one-year mortality; furthermore, extended hospitalizations, heightened demand for intensive care, and hemodialysis resulting from CIN occurrence impose an extra financial burden on healthcare expenditures [22].

Timely identification of high-risk patients is essential to ensure appropriate risk stratification and careful monitoring of vital signs and fluid status. Several factors, such as congestive heart failure, renal impairment, advanced age, female sex, and DM, are well-established risk factors for CIN [23-26]. These variables are also components of the CHA₂DS₂-VASc score [27]. The findings of this study indicate that the CHA₂DS₂-VASc score can serve as a strong predictor of CIN, highlighting its potential as a simple, cost-effective, and practical tool for pre-procedural risk assessment.

In this study, patients with higher CHA₂DS₂-VASc scores were generally older and had a greater burden of cardiovascular risk factors, particularly HTN and diabetes, as well as more frequent use of ACE inhibitors, ARBs, and metformin compared to those with lower scores. A similar higher proportion of HTN and diabetes was reported by Bhat et al. (2025) [18]. A significant association between high CHA₂DS₂-VASc and the development of CIN was found. In the present study, 13.5% of participants developed CIN following PCI. This incidence is comparable to previous reports in the literature. For instance, a study reported CIN in 14.3% of patients undergoing PCI for ACS [18], while Chaudhary et al. [16] observed a similar rate of 13.7%. Similarly, Kurtul et al. [27] and Wang et al. [14] documented incidences of 11.3% and 16.3%, respectively, aligning closely with the findings of the present study. In contrast, Cicek and Yıldırım [28] reported a higher incidence of 23.3%, whereas Kumar et al. [29] found a lower incidence of 9.1%. Collectively, these findings suggest that the incidence of CIN in the current study is consistent with previously published international data, supporting the reliability and generalizability of the observed rate within the expected range for patients undergoing PCI.

In the present study, the development of CIN was not significantly associated with age or sex. This finding is consistent with the study by Chaudhary et al. (2019) [16], who also reported no statistically significant difference in CIN incidence across age groups. However, Bhat et al. (2025) [18] observed a trend toward increased CIN incidence with advancing age, a result that corresponded with the findings of Wang et al. (2019) [14] and Kumar et al. (2021) [29], who similarly noted higher susceptibility among older patients. In contrast, Bhat et al. [18] found no significant association between CIN and sex, which is in agreement with the present study. While older age may predispose to renal vulnerability in some populations, the impact of age and sex on CIN risk may vary depending on patient characteristics, baseline renal function, and procedural factors across different study cohorts.

In this study, 86.5% of the study participants were suffering from multiple comorbidities. However, no association between CIN and multiple comorbidities was noted. However, a study conducted by Bhat et al. (2025) [18] found a significant association between multiple comorbidities and CIN. Comorbidities like HTN and DM were found to be significantly associated with CIN, as reported by several studies [14,18,27-29]. In the present study, HTN showed a significant association with the development of CIN, whereas no such relationship was observed with DM, dyslipidemia, history of vascular disease, or the volume of contrast media used. Conversely, numerous prior studies have indicated substantial correlations between CIN and CAD and the volume of contrast administered [16,18,28]. The discrepancy may be attributed to differences in patient selection, baseline renal function, or variations in procedural techniques and contrast protocols, which could lead to different outcomes in the incidence of CIN across studies.

In this study, patients with a CHA₂DS₂-VASc ≥2 were found to have more than a fivefold increased risk of developing CIN. This finding is consistent with that of Rantung et al. (2025), who reported nearly a fourfold higher risk among patients with a score of ≥2.5 [19]. Similar associations were observed by Samir et al. [11] and Abdel-Ghany et al. [30], reinforcing the predictive value of this scoring system. Chaudhary et al. (2019) [16] also demonstrated that a CHA₂DS₂-VASc score ≥4 was linked to more than a twofold increase in CIN risk. The variation in the magnitude might be due to the difference in study design, sample size, characteristics of the participants, and baseline renal function, which are among the most influential contributors. Additionally, the reported incidence and strength of association may also be influenced by differences in contrast type and volume, hydration protocols, concomitant nephrotoxic drugs, and timing of post-procedural creatinine measurement. Therefore, while all studies consistently demonstrate a positive correlation between elevated CHA₂DS₂-VASc scores and CIN, the variation in odds ratios likely reflects heterogeneity in patient populations, procedural factors, and methodological approaches. Taken together, these findings emphasize the diagnostic value of the CHA₂DS₂-VASc score beyond its traditional use in atrial fibrillation.

The CHA₂DS₂-VASc score ≥2 demonstrated moderate sensitivity (78.57%) but low specificity (54.44%) in predicting CIN, indicating a reasonable ability to identify at-risk patients, albeit with a higher rate of false positives. These findings are generally consistent with previous studies, although some differences in diagnostic performance were observed. For instance, Bhat et al. (2025) reported higher sensitivity (92.3%) and specificity (75.6%) at a cutoff value of ≥2, suggesting stronger predictive performance in their cohort [18]. Similarly, Chaudhary et al. (2019) demonstrated sensitivity of 90.2% and specificity of 62.9% using the CHA₂DS₂-VASc score, with variations observed at higher cutoff thresholds [16]. These differences may reflect heterogeneity in study populations, sample size, baseline risk profiles, and methodological approaches across studies. The CHA₂DS₂-VASc score is a reliable screening measure for identifying patients at risk of CIN, as evidenced by its consistently high sensitivity across trials.

The CHA₂DS₂-VASc score can predict CIN using readily accessible clinical factors, highlighting its practical relevance as a bedside risk assessment tool before PCI. Early identification of high-risk patients enables personalized preventative treatments, such as proper hydration and cautious procedural planning, which improves patient safety. The present findings are consistent with earlier investigations, which adds to the external validity of this grading system. Incorporating the CHA₂DS₂-VASc score into pre-PCI evaluations can improve renal outcomes and clinical management.

Limitations

The study was conducted in a tertiary care hospital with a relatively small sample size, which limits the generalizability of the study findings. One limitation of this study relates to the structure of the CHA₂DS₂-VASc score in this cohort. As all PCI patients had vascular disease, each received at least one point, restricting the CHA₂DS₂-VASc <2 group to those without additional risk factors. This may have introduced some imbalance between groups, and the observed association with CIN may partly reflect the burden of comorbidities rather than the independent predictive ability of the score. Due to the nature of the study design, the causal relationship between CHA₂DS₂-VASc and CIN could not be established. In addition, the relatively small number of CIN events may have limited the robustness of the multivariate logistic regression analysis, potentially resulting in model instability and wide CIs. Several factors, such as hydration status, type and osmolality of contrast media, timing of exposure to nephrotoxic drugs, and peri-procedural hemodynamic fluctuations, were not measured, which might have influenced the prediction. Additionally, serum creatinine was measured only up to 48 hours post-procedure, which may have underestimated late-onset CIN cases. Finally, receiver operating characteristic (ROC) curve analysis and area under the curve (AUC) estimation were not performed, which may limit a comprehensive assessment of the overall discriminative performance of the CHA₂DS₂-VASc score.

## Conclusions

In this study, CIN occurred in over 13% of patients undergoing PCI. Patients with a CHA₂DS₂-VASc score ≥2 had more than fivefold increased odds of developing CIN, establishing the score as an independent and valid predictor. Owing to its simplicity, cost-free nature, and reliance on routinely available clinical variables, the CHA₂DS₂-VASc score may serve as an effective pre-procedural tool for early risk stratification of CIN, particularly in resource-constrained settings. Notably, the score is especially useful for ruling out CIN and is better suited as a screening rather than a confirmatory tool. Incorporation of this scoring system into routine clinical assessment may aid in identifying high-risk patients who require closer monitoring, optimal hydration, and preventive strategies to mitigate post-PCI renal complications. Further multicenter studies with larger sample sizes and longer follow-up are recommended to validate these findings and refine predictive thresholds in the Bangladeshi population.
